# Artificial intelligence models in the surgical planning of low-grade gliomas: a systematic review

**DOI:** 10.3389/fonc.2025.1672289

**Published:** 2026-01-15

**Authors:** Vivek Sanker, Aparna Venkatesan, Afia Salma, Ajeya Sp, Zhikai Li, Philip Heesen, Chelsea Park, David J. Park, Atman Desai

**Affiliations:** 1 Department of Neurosurgery, Stanford University, Palo Alto, CA, United States; 2 Department of Neurosurgery, Government Medical College, Omandurar, Government Estate, Chennai, India; 3 Department of Neurosurgery, Dow University of Health Sciences, Karachi, Pakistan; 4 Department of Medicine, Indira Gandhi Medical College and Research Institute, Puducherry, India; 5 Department of Clinical Neurosciences, Addenbrooke’s Hospital, Cambridge, United Kingdom; 6 Department of Neurosurgery, University of Zurich, Zurich, Switzerland

**Keywords:** low-grade glioma, artificial intelligence, machine learning, predictive models, surgical resection

## Abstract

**Introduction:**

AI techniques like convolutional neural networks (CNN), deep learning (DL), and neural networks (NN) have made it easier to automatically extract important clinical data for glioma post-treatment monitoring and surgical planning.

**Objective:**

To systematically review and analyze the role of AI/ML models in the surgical planning of LGG.

**Methodology:**

A rigorous and comprehensive systematic literature search was conducted across PubMed, Scopus, Web of Science Advance, ArXiV, and Embase (Ovid) databases from inception to July 14, 2025. Articles related to the utility of ML models in the surgical planning of LGG were included.

**Results:**

Our review included eight studies in both preoperative and intraoperative settings with variation in the type of AI applied, such as tumor segmentation, intraoperative neuro navigation, hyperspectral imaging, and surgical recommendation. Upon comparative analysis of mean DICE coefficients of the proposed models for segmentation, the DeepMedic CNN was found to have the highest DICE for tumor segmentation. With hyperspectral imaging, the use of MLP classifiers yields high accuracy; however, when taking into consideration the quality of tiles, DL methods outperform the classical methods by ~10%. Survival Probability using the Balanced Survival lasso-network (BSL), balanced individual treatment effect (BITES), and DeepSurv models: Difference in restricted mean survival time (DRMST) between the Consis group and In-consis group [4.75 (1.54-7.95)] for BSL, [3.81 (0.63–6.98)] for Deep Surv, and [3.76 (0.57–6.96)] for BITES.

**Conclusions:**

AI/ML models have shown promising results in diagnostic and management approaches for glioma resection. Nonetheless, this is based on a small number of studies (n=8) and remain preliminary. Validating the findings in external datasets with a larger patient population would help enhance the predictive capacity of the existing models.

## Introduction

Low-grade gliomas are primary brain tumors with a neuroepithelial origin that often occur in the younger demographic. These slow-growing tumors usually have a better prognosis than malignant high-grade gliomas, however, they have the potential to grow more aggressively within decades (1, 2). Classified by WHO as Grade 1 or 2, this group of tumors are of a diverse nature and are hallmarked by a lack of (grade 1) to minimal cytological atypia (grade 2) on histopathological findings. The 2016 update to the WHO classification added the inclusion of molecular markers, while the 2021 update introduced the grading of tumors within categories, instead of solely across (3–5).

Due to their indolent nature, early age of presentation and heterogeneity, low-grade gliomas are challenging to both diagnose and treat, with several factors needing to be considered – in particular, the molecular classification (such as IDH-type mutations and 1p/19q co-deletion) and frequent involvement of eloquent areas (1, 6). Other factors of importance include the age of the patient and due consideration for their wishes on matters such as fertility (7). Surgical treatment is the mainstay for tissue biopsy and resection; the former to establish the molecular makeup of the neoplasm and the latter ranging from extensive resection to debulking. However, the goal of surgical treatment is generally to prevent or interrupt an aggressive transformation or progression (8). Survival prediction of patients is often impacted by surgical factors such as the extent of resection (9, 10).

Surgical management of low-grade gliomas poses unique challenges, particularly in the preoperative and intraoperative phases. Preoperative imaging is critical for defining tumor extent, relationship to eloquent cortex, and white matter tract involvement, which directly influence surgical approach and resection goals (11, 12). Intraoperatively, real-time navigation and tissue differentiation remain challenging despite advances in neuromonitoring and intraoperative imaging (13). The balance between maximizing extent of resection while preserving neurological function requires sophisticated tools to assist neurosurgeons in decision-making during these critical phases.

Artificial intelligence (AI) and machine learning techniques have emerged as investigational tools in neuro-oncology, with early research exploring their potential applications in image analysis, tumor segmentation, and outcome prediction (13, 14). While these technologies show promise in research settings for tasks such as automated tumor delineation on MRI/CT scans, molecular subtype prediction from imaging features, and simulation of white matter tractography, they remain predominantly in the validation and development stage without widespread clinical deployment (14). Current AI applications in low-grade gliomas are primarily focused on preoperative imaging analysis to aid surgical planning and intraoperative imaging interpretation to guide resection extent (13, 14, 33). However, the clinical validity, generalizability, and practical implementation of these tools require systematic evaluation before routine clinical integration.

The goal of this systematic review is to comprehensively evaluate the current state of artificial intelligence and machine learning applications specifically in preoperative and intraoperative imaging for low-grade glioma surgery. We aim to synthesize the evidence on AI-based tools for tumor segmentation, molecular classification prediction, tractography, and surgical navigation, assess their performance metrics and clinical utility, and identify the limitations and gaps in current research that must be addressed before clinical translation.

## Materials and methods

### Ethical review

Ethical review and approval were waived for this study as it is a systematic review of previously published literature and did not involve direct human participation or the collection of primary clinical data.

### Search strategy

A comprehensive and systematic search was conducted across PubMed, Scopus, Web of Science Advance, ArXiV, and Embase (Ovid) to identify relevant studies assessing the use of artificial intelligence (AI) and machine learning (ML) models in the surgical planning of low-grade gliomas (LGGs). The search spanned from database inception to July 14, 2025. Search terms included combinations of keywords and MeSH/Emtree terms such as ‘artificial intelligence’, ‘surgical approaches’, ‘low-grade glioma’, and ‘eloquent cortex’ (see **Supplementary Table 1** for full search syntax across databases).

Inclusion criteria comprised studies involving adult patients diagnosed with LGG that applied AI/ML techniques in preoperative or intraoperative surgical settings. Exclusion criteria encompassed studies focused solely on high-grade gliomas, purely radiological applications without surgical relevance, reviews, commentaries, editorials, and non-human/animal research. No restrictions were placed on study design, publication language, or geographic location.

### Screening of studies

**Figure 1:**
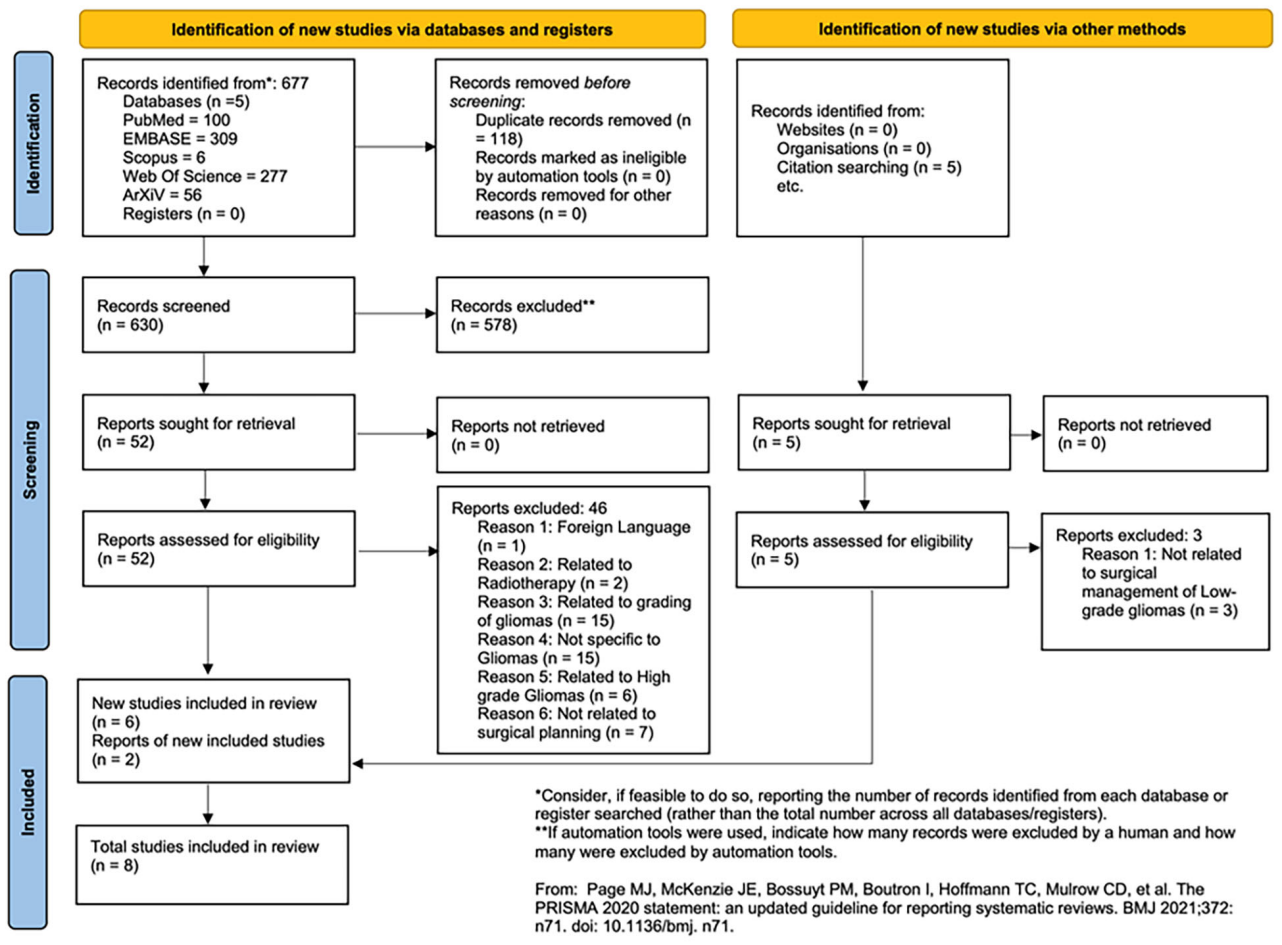
PRISMA flow diagram. A PRISMA flow diagram is presented to illustrate the study selection process, including identification, screening, eligibility, and inclusion.

Initial screening was performed independently by two reviewers (AV and AS) based on titles and abstracts. Eligible articles were then subjected to full-text screening for inclusion based on predefined criteria. Any discrepancies were resolved through consensus with a third reviewer. The screening process was conducted in accordance with the PRISMA 2020 guidelines, with the study selection flow summarized in the PRISMA diagram (**Figure 1**).

### Data extraction

Data extraction was independently conducted by two reviewers (AV and AS) using a standardized form. Extracted variables included study design, year of publication, sample size, type of AI/ML model utilized, domain of application (e.g., tumor segmentation, neuro-navigation, hyperspectral imaging), performance metrics (e.g., Dice coefficient, classification accuracy, survival prediction models), and reported clinical impact. Discrepancies were reconciled through discussion and consensus.

### Data analysis

Quantitative data from model performance was analyzed, focusing on segmentation accuracy, classification precision, and survival prediction outcomes. The Dice coefficient, accuracy percentage, and restricted mean survival time (RMST) were compared across studies employing different algorithms (e.g., DeepMedic CNN, MLP classifiers, BITES, DeepSurv). Where appropriate, weighted averages and ranges were computed. Statistical summarization was performed using Microsoft Excel, R Statistical Software (v4.3.1), and Python (v3.13.3).

### Quality assessment

The methodological quality and risk of bias of the included studies were evaluated using the PROBAST (Prediction model Risk of Bias Assessment Tool), as applied to each AI model’s design and validation framework (**Supplementary Table 1**). PROBAST assessed four key domains:

- Participants: Representativeness and clinical relevance of data sources and training populations.
- Predictors: Clarity and consistency of input data and variables.
- Outcome: Definition and appropriateness of model endpoints.
- Analysis: Statistical rigor, validation approach, and bias management.

## Results

677 articles were identified from searching 5 databases. After duplicate removal, 630 records were screened, 52 full texts were screened, and 6 were included in the review. After citation searching, 5 records were identified and their full texts assessed for eligibility. Of these, another 2 reports were included in the review for a total of 8 articles (**Figure 1**).

### Overview of included studies

The eight included studies span preoperative and intraoperative domains, collectively involving patients with low-grade gliomas, with some studies extending to pediatric or broader glioma cohorts. Four studies evaluated AI/ML applications in preoperative settings, three focused exclusively on intraoperative modalities, and one addressed both contexts. Applications included tractography, tumor segmentation, neuro-navigation, optical biopsy, and surgical decision modeling. Details regarding the included studies, segmentation model, intraoperative AI, and surgical recommendation models are provided (**Tables 1**–**4**).

**Table 1:** Review overview.

Study	Cohort size	AI application domain	Setting
Lucena et al. (2023) (15)	15 patients	Tractography (UncSeg with nTMS validation)	Preoperative
Zhu et al. (2023) (16)	842 patients	Surgical recommendation via causal inference	Preoperative
Kazerooni et al. (2023) (17)	244 patients	Tumor & brain segmentation with DeepMedic CNN	Preoperative
Vafaeikia et al. (2022) (18)	Not reported	Tumor segmentation via deep multitask learning	Preoperative
Sun et al. (2016) (19)	79 patients	Functional neuronavigation with intraoperative MRI & AR	Intraoperative
Jermyn et al. (2015) (20)	Not reported	Raman spectroscopy-based margin detection	Intraoperative
Giannantonio et al. (2024) (21)	5 patients	Hyperspectral imaging with DL channel reduction	Intraoperative
Black et al. (2024) (22)	184 patients	Hyperspectral classification, IDH/margin prediction	Both

Kazerooni et al. included pediatric LGGs, while Zhu et al. employed SEER registry data. Vafaeikia cohort size not specified.

**Table 2:** Segmentation model performance.

Study	Model	Target region(s)	Dice coefficient (Mean ± SD)
Kazerooni et al.	DeepMedic CNN	Whole tumor	0.91 ± 0.10
		Enhancing tumor	0.73 ± 0.27
		Non-enhancing components (NET/CC/ED)	0.79 ± 0.19
		Brain tissue (skull stripping)	0.98 ± 0.02
Lucena et al.	UncSeg	Corticospinal tract (CST) vs nTMS response	High OC; Negative uncertainty correlation
Vafaeikia et al.	dMTL Network	Tumor segmentation with genetic classifier	Improved over baseline (no raw Dice reported)

Lucena et al. did not report Dice scores but quantified overlap coefficients and uncertainty metrics.

Weighted Average Dice (whole tumor): *0.905 (Kazerooni dataset only).*

**Table 3:** Intraoperative AI performance.

Study	Model type	Target/Outcome	Metric/Accuracy
Sun et al.	Augmented reality + neuronavigation	Extent of resection (EOR) and functional preservation	EOR: 95.2%; Motor/language function significantly better (p < 0.01)
Jermyn et al.	Raman spectroscopy	Cancer cell detection at margins	Sensitivity: 93%; Specificity: 91%
Giannantonio et al.	DL with HSI channel reduction	Tumor demarcation	5-patient demo; high accuracy reported qualitatively
Black et al.	MLPs/Random Forests	Tumor grade, IDH status, margin class	Accuracy: 84–96.1%; p < 0.01 fluorophore differences

Only Black et al. reported formal test set accuracies per classifier.

**Table 4:** Surgical recommendation models.

Study	Model	Output	Metric
Zhu et al.	DL + causal inference	STR vs GTR recommendation	DRMST: BSL (4.75 mo), DeepSurv (3.81 mo), BITES (3.76 mo)
		Grouped by recommendation adherence	Consis. group had better BCSS

DRMST, Difference in Restricted Mean Survival Time; BITES, Balanced Individual Treatment Effect; BSL, Balanced Survival Lasso.

### Segmentation model performance

Among the four studies focused on **segmentation**, the most consistently reported metric was the **Dice coefficient**, which evaluates overlap between predicted segmentation and ground truth.

### Intraoperative classification and surgical utility

Three studies (plus Black et al.) focused on **real-time classification**, intraoperative **margin delineation**, or **resection enhancement**. Classification accuracy and preservation of neural function were key outcomes.

### Surgical recommendation and causal modeling

Zhu et al. employed deep learning and causal inference to predict individual survival benefits based on surgical strategy.

## Discussion

This systematic review synthesizes evidence on the application of artificial intelligence (AI) and machine learning (ML) technologies in the surgical management of low-grade gliomas (LGGs), with a particular emphasis on their role in anatomically eloquent regions. In synthesizing the included studies, three thematic domains emerged: preoperative segmentation and planning, intraoperative classification and guidance, and hybrid diagnostic-operative platforms. Preoperative models such as DeepMedic CNN achieved high segmentation accuracy (Dice 0.91 for whole tumor), while tractography tools like UncSeg aligned with functional mapping, supporting their relevance in anatomically eloquent regions. Intraoperative tools—including Raman spectroscopy and hyperspectral imaging—demonstrated strong diagnostic precision (sensitivities >90%), and augmented reality neuronavigation improved both extent of resection and postoperative outcomes. Causal inference frameworks predicted survival benefit based on surgical strategy, underscoring AI’s potential in personalized decision-making. Collectively, these findings highlight the technical feasibility and emerging clinical utility of AI-enhanced workflows across the LGG surgical continuum.

Preoperative applications concentrated on tumor and tissue segmentation, as well as surgical strategy personalization. DeepMedic CNN, as employed by Kazerooni et al., demonstrated high Dice similarity scores for whole tumor segmentation (0.91 ± 0.10) and brain tissue extraction (0.98 ± 0.02), supporting the feasibility of automated volumetric mapping in pediatric gliomas (17). Meanwhile, Lucena et al.’s tractography model (UncSeg) aligned DL-generated corticospinal tract predictions with nTMS motor response masks, revealing the capacity of uncertainty estimates to reflect anatomical validity in distorted surgical fields (15). Vafaeikia et al. advanced this concept by incorporating genetic classifiers into a multitask segmentation pipeline, leveraging molecular signatures to refine pediatric LGG localization (1, 18). However, these segmentation models were primarily validated on internal datasets, with limited cross-institutional testing. This raises concerns about generalizability, particularly in anatomically distorted or heterogeneous LGG presentations.

Zhu et al.’s large-scale causal modeling study exemplifies how ML frameworks can inform surgical choices. Their approach, integrating Balanced Survival Lasso (BSL), DeepSurv, and BITES models, quantified survival advantages based on extent of resection and treatment adherence. Patients whose clinical management aligned with algorithmic predictions experienced significantly improved brain cancer-specific survival, reinforcing the utility of AI in resolving therapeutic ambiguity (13, 16). Yet, the reliance on retrospective registry data and absence of prospective validation limits the immediate clinical applicability of these recommendations.

Intraoperative modalities included a diverse array of imaging and classification tools. Jermyn et al. validated the use of Raman spectroscopy for real-time cancer cell detection at surgical margins, achieving sensitivity and specificity rates of 93% and 91%, respectively (20). Hyperspectral imaging (HSI) platforms, explored by Black et al. and Giannantonio et al., exploited fluorophore abundance profiles and neural network channel optimization to distinguish tumor boundaries and molecular traits intraoperatively, with classification accuracies exceeding 96% (21, 22). Sun et al. demonstrated that pairing augmented reality with intraoperative MRI and functional neuronavigation yields substantial gains in extent of resection (95.2%) and postoperative functional outcomes (p < 0.01), highlighting the clinical value of AI-supported intraoperative visualization (13, 19). Nevertheless, such high accuracies, while promising, may reflect early-phase optimism. Without benchmarking against intraoperative gold standards or reporting of failure cases, the robustness of these tools in live surgical environments remains uncertain.

Black et al.’s dual-context implementation bridges the diagnostic-to-operative continuum, underscoring the future potential of integrated AI platforms that unify planning, execution, and evaluation in glioma resection (22). Collectively, these findings suggest that AI models, when rigorously trained and validated, can offer neurosurgeons enhanced anatomical insight, decision support, and operative precision, particularly in cases where functional preservation is critical.

It is nonetheless crucial to reiterate that several studies often report extremely high classification accuracies (often exceeding 95%) without rigorous clinical benchmarking against gold standards or failure modes. This may reflect early-phase optimism rather than validated clinical efficacy (23). There is also a literature gap in evaluating how these tools influence real-time surgical decision making particularly in anatomically complex or eloquent regions (24–26). Transitioning from proof-of-concept demonstrations to clinical utility, future research must prioritize external validation across diverse cohorts, model explainability to enhance clinician trust, real-world integration studies involving neurosurgical teams, and regulatory collaboration to meet acceptable standards for approval and wide deployment (12, 13, 27).

Furthermore, many models, particularly deep learning architectures, function as ‘black boxes’ with limited interpretability, which may hinder clinician trust and regulatory approval. Furthermore, most studies relied on internal validation, with few demonstrating reproducibility across independent cohorts or institutions. These gaps underscore the need for transparent, explainable AI frameworks and robust external validation pipelines.

### Limitations

Despite promising advances and future trajectories highlighted above, several key limitations still exist that currently hinder wide clinical adoption of these AI tools.

First, there was considerable heterogeneity in data reporting across the included studies. Variations in cohort size, imaging modalities, and outcome measures posed challenges to direct comparison, with some studies omitting key performance indices such as confidence intervals or raw Dice coefficients. This inconsistency limited the ability to perform a robust quantitative synthesis.

Second, the scope of patient populations was uneven. Two of the included studies focused exclusively on pediatric LGGs, raising concerns about the generalizability of their findings to adult populations, particularly given the differing radiographic characteristics and molecular profiles between these groups (28, 29).

Third, the validation of AI models was largely confined to internal datasets. The predominant reliance on internal validation raises concerns about reproducibility and generalizability, which are essential for regulatory approval and clinical integration. External validation on independent cohorts, or deployment in prospective clinical settings, was rarely undertaken, thereby reducing confidence in the models’ translational applicability (30, 31). The lack of cross-institutional reproducibility highlights the need for broader testing before integration into clinical workflows.

Fourth, many AI models function as ‘black boxes’, particularly deep learning frameworks, offering limited interpretability to clinicians (27, 32). This hinders trust in automated recommendations and impedes regulatory approval.

Fifth, several studies featured limited sample sizes or exploratory demonstration datasets, thus undermining statistical robustness and impeding the development of clinically deployable algorithms. For instance, Giannantonio et al.’s hyperspectral imaging model was tested in only five patients (21), while Vafaeikia et al. did not report cohort size at all (18). These constraints illustrate the early-stage nature of certain approaches and highlight the need for larger, multicenter evaluations.

Finally, while technical metrics such as segmentation accuracy and classification precision were well documented, the integration of downstream clinical outcomes, such as recurrence rates, survival beyond restricted mean survival time, and long-term functional preservation, was inconsistent across studies. This gap restricts the ability to draw firm conclusions about the real-world impact of AI-assisted neurosurgical planning.

Taken together, these limitations underscore the challenges in translating technical advancements into routine surgical care. Addressing these gaps will require collaboration between clinicians, data scientists, and regulatory bodies to improve transparency, scalability, and clinical alignment.

## Conclusion

Artificial intelligence and machine learning models represent a paradigm shift in the neurosurgical management of low-grade gliomas, offering tools that refine preoperative planning, enhance intraoperative precision, and guide personalized treatment strategies. Segmentation algorithms such as DeepMedic CNN (Dice 0.91), tractography models like UncSeg, and intraoperative classifiers including Raman spectroscopy and hyperspectral imaging demonstrated promising technical reliability and diagnostic utility, particularly in anatomically complex cases.

However, the evidence remains preliminary, with most models validated only on internal datasets and limited sample sizes. Prospective validation, multicenter trials, and regulatory frameworks are essential to ensure safe and equitable integration into practice. As computational intelligence becomes increasingly embedded in clinical workflows, it may redefine neurosurgical standards, fostering a future of precision-guided brain tumor care.

## Data Availability

The original contributions presented in the study are included in the article/**Supplementary Material**. Further inquiries can be directed to the corresponding author.
