# Supplementary material for: Artificial intelligence models in the surgical planning of low-grade gliomas: a systematic review
Source: Front Oncol. 2026 Jan 15;15:1672289. doi: 10.3389/fonc.2025.1672289 (PMC12851994; doi:10.3389/fonc.2025.1672289)
Supplement: Supplementary file 1 [file Table1.docx]

**Supplementary Materials**

**Search Strategy**

| **Database** | **No** | **Search Query** | **Results** | |
| --- | --- | --- | --- | --- |
| **EMBASE** | | | | |
|  | #1 | #2 AND 'article'/it 309  #1 (('artificial intelligence'/exp OR 'artificial intelligence' OR ai) AND ('surgical approach'/exp OR 'surgical approach' OR 'neurosurgery'/exp OR neurosurgery) AND 'brain eloquence' OR 'eloquent areas') AND ('low-grade glioma'/exp OR 'low-grade glioma' OR lgg) 542 | 309 | |
| **PubMed** | | | | |
|  | #1 | ((("artificial intelligence"[All Fields] OR ("antagonists and inhibitors"[MeSH Subheading] OR ("antagonists"[All Fields] AND "inhibitors"[All Fields]) OR "antagonists and inhibitors"[All Fields] OR "ai"[All Fields])) AND ("surgical approach"[All Fields] OR ("neurosurgery"[MeSH Terms] OR "neurosurgery"[All Fields] OR "neurosurgeries"[All Fields] OR "neurosurgery s"[All Fields] OR "neurosurgical procedures"[MeSH Terms] OR ("neurosurgical"[All Fields] AND "procedures"[All Fields]) OR "neurosurgical procedures"[All Fields]))) OR ("brain eloquence"[All Fields] OR "eloquent areas"[All Fields])) AND ("low-grade glioma"[All Fields] OR "LGG"[All Fields]) | 100 | |
| **Scopus** | | | | |
|  | #1 | ( TITLE-ABS-KEY ( "artificial intelligence" ) OR TITLE-ABS-KEY ( ai ) ) AND ( TITLE-ABS-KEY ( "surgical approach" ) OR TITLE-ABS-KEY ( "neurosurgery" ) ) OR ( TITLE-ABS-KEY ( "brain eloquence" ) OR TITLE-ABS-KEY ( "eloquent areas" ) ) AND ( TITLE-ABS-KEY ( "low-grade glioma" ) OR TITLE-ABS-KEY ( lgg ) ) | 6 | |
| **Web of Science** | | | | |
|  | #1 | TS=("artificial intelligence" OR AI) AND TS=("surgical approach" OR neurosurgery) OR TS=("brain eloquence" OR "eloquent areas") AND TS=("low-grade glioma" OR LGG) and Article (Document Types) and English (Languages) | | 277 |
| **ArXiV** | | | | |
|  | #1 | (Low grade glioma) | | 56 |
